# The Development of a Clinical Registry Digital Database on Invasive Fungal Infections in India: Advancing Epidemiological Understanding and Patient Care

**DOI:** 10.3390/jof10010042

**Published:** 2024-01-05

**Authors:** Harleen Kaur, Kh. Jitenkumar Singh, Saurabh Sharma, Madhuchhanda Das, Venencia Albert, Anup Kumar Ojha, Gagandeep Singh, Vinaykumar Hallur, Jayanthi Savio, Umabala Pamidimukkala, Tadepalli Karuna, Reema Nath, Immaculata Xess, Prashant Gupta, Anjali Shetty

**Affiliations:** 1 ICMR-National Institute of Medical Statistics (ICMR-NIMS), New Delhi 110029, India; harleenkr.92@gmail.com (H.K.); jitensinghkh@gmail.com (K.J.S.); 2 Indian Council of Medical Research (ICMR), New Delhi 110029, India; albertvenencia.icmr@gmail.com (V.A.); ojhaicmr@gmail.com (A.K.O.); 3 Department of Microbiology, All India Institute of Medical Sciences (AIIMS), New Delhi 110029, India; drgagandeep@gmail.com (G.S.); immaxess@gmail.com (I.X.); 4 Department of Microbiology, All India Institute of Medical Sciences (AIIMS), Bhubaneswar 751019, India; vinay118@gmail.com; 5 Department of Microbiology, St. John’s Medical College (SJMC), Bengaluru 560034, India; jayanthisjmc@gmail.com; 6 Department of Microbiology, Nizam’s Institute of Medical Sciences (NIMS), Hyderabad 500082, India; umapamidi@gmail.com; 7 Department of Microbiology, All India Institute of Medical Sciences (AIIMS), Bhopal 462020, India; karuna.microbiology@aiimsbhopal.edu.in; 8 Department of Microbiology, Assam Medical College (AMC), Dibrugarh 786002, India; reemanath.assam@gmail.com; 9 Department of Microbiology, King George’s Medical University (KGMU), Lucknow 226003, India; prashantgupta46@hotmail.com; 10 PD Hinduja, Mumbai 400016, India; dr_ashetty@hindujahospital.com

**Keywords:** mycology, invasive fungal infections, diagnosis, epidemiology, clinical registry, case report form, public health

## Abstract

A well-structured digital database is essential for any national priority project as it can provide real-time data analysis and facilitate quick decision making. In recent times, particularly after the COVID-19 pandemic, invasive fungal infections (IFIs) have emerged as a significant public health challenge in India, affecting vulnerable population, including immunocompromised individuals. The lack of comprehensive and well-structured data on IFIs has hindered efforts to understand their true burden and optimize patient care. To address this critical knowledge gap, the ICMR has undertaken a Pan-India pioneer initiative to develop a network of Advanced Mycology Diagnostic research centres in different geographical zones of the country (ICMR-MycoNet). Under the aegis of this project, a clinical registry on IFIs in the ICUs is initiated. This process paper presents a detailed account of the steps involved in the establishment of a web-based data entering and monitoring platform to capture data electronically, ensuring robust and secure data collection and management. This system not only allows participating ICMR-MycoNet centres to enter patient information directly into the database using standardized Case Report Form (CRF) but also includes data validation checks to ensure the accuracy and completeness of entered data. It is complemented by a real-time, web-based, and adaptable data visualization platform. This registry aims to provide crucial epidemiological insights, promote evidence-based hospital infection control programs, and ultimately improve patient outcomes in the face of this formidable healthcare challenge.

## 1. Introduction

A real-time analytical database is crucial for research data management focussed on national priority problems. It plays a pivotal role in providing policymakers and researchers with timely insights into data trends prior to the completion of the project, enabling a more informed assessment of progress and impact. In recent times, fungal diseases have attracted great attention due to COVID-Associated Pulmonary Aspergillosis (CAPA) and COVID-Associated Mucormycosis (CAM). During the second wave, CAM was prioritized and declared as a notifiable disease by the Ministry of Health, Government (Govt.) of India. Overall, fungal pathogens cause a minimum of 13 million infections and 1.5 million fatalities worldwide each year, mainly affecting individuals with compromised immune systems [1,2,3,4,5]. This has led to growing concern among global experts who predict that fungal outbreaks may present a significantly greater threat to humanity in the near future, surpassing what was previously perceived [6,7,8,9].

In recent years, India has witnessed a significant rise in the incidence of IFIs, mirroring global trends. The increased prevalence of immunocompromising conditions, such as HIV/AIDS, cancer, organ transplantation, and the widespread use of immunosuppressive therapies, have contributed to this alarming phenomenon [6,10,11]. Another group is that of the non-immunocompromised individuals who are critically ill and have long-term hospital stays. Pathogens such as *Candida* spp., *Aspergillus* spp., *Cryptococcus neoformans*, and *Mucorales* are responsible for severe IFIs, leading to increased healthcare burden and substantial challenges in patient management. An additional cause of concern is the worldwide emergence of multi-drug-resistant fungal strains, which significantly worsens treatment outcomes and increases mortality rates. Many fungal species have developed resistance to all four classes of antifungal drugs: polyenes, azoles, echinocandins, and the pyrimidine analogue 5-flucytosine [12]. A recent systematic review published by Ray et al. also showed that burden of fungal infection is high (4.1%) in India, but unrecognized, due to the lack of surveillance data [13].

Clinical database development is a crucial aspect of modern healthcare and medical research. The quality of medical care is not solely dependent on the knowledge of disease processes but also on how the practitioners collect, organize, and interpret the clinical information to diagnose and treat diseases effectively and efficiently. For example, The Cancer Genome Atlas (TCGA); Gene Expression Omnibus (GEO); Surveillance, Epidemiology and End Results (SEER); Medical Information Mart for Intensive Care (MIMIC); and others are a few databases that can facilitate increased global cooperation to promote clinical practice, education, and scientific research that can aid in the decision-making process in precision medicine, and the emergence of a new health management model [14]. With the increasing prevalence of fungal infections and the associated morbidity and mortality rates, effective data management plays a pivotal role in understanding the epidemiology, risk factors, treatment outcomes, and the development of evidence-based interventions. Although several fungal pathogen databases and registries are available globally, the majority of them are focused on basic research. For example, the *Candida* Genome Database (CGD) specializes in *Candida*, a fungal pathogen causing human opportunistic infections, offering genomic data and functional annotations for *Candida* species [15], while the Fungal Multi-Locus Sequence Typing Database (MLST) assesses fungal pathogen genetic diversity [16]. FungiScope™ and Fungal India Registry (Fung-I-Reg) are other database registries operating across the globe focussing on various species [16,17,18,19,20,21]. Data management reduces manual error, cost, and time, especially for multicentre research studies. By harnessing the information provided by these databases and registries, researchers and healthcare professionals can enhance their understanding of fungal infections and epidemiological patterns, detect emerging threats, develop targeted treatments, and improve patient outcomes. Additionally, such resources can also aid in formulating public health policies and guidelines for the prevention and control of fungal infections. 

In the absence of a comprehensive clinical registry database, data on IFIs in India have been limited to small-scale studies and institutional databases, making it difficult to grasp the true magnitude of the problem and implement effective strategies for disease management and prevention. To address these shortcomings, healthcare experts, researchers, and policymakers united to establish a clinical registry database on IFIs in India.

This process paper provides a detailed account of the key steps involved in the development of the registry database, from conceptualization to implementation and beyond. It highlights the importance of a national registry and also discusses the challenges involved in developing and implementing a database in resource-poor countries, along with the strategies to sustain a database.

## 2. Materials and Methods

**Figure 1:**
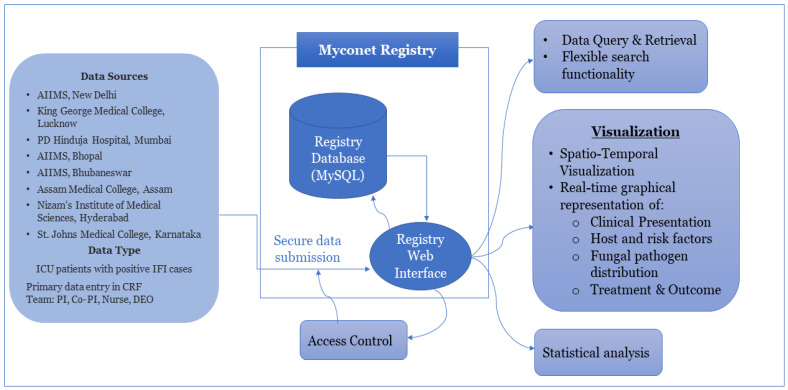
Flow diagram depicting the database registry developed for the collection, storage, and dissemination of the MycoNet data (https://clinicomycoregistry.icmr.org.in/, accessed on 2 November 2023).

The ICMR is an autonomous apex research body under the Ministry of Health and Family Welfare, Government of India, which works on the formulation, coordination, and promotion of biomedical research. The ICMR advanced mycology laboratories (ICMR-MycoNet) was a network initiated in 2019 under a national task force project and systematically expanded to a total of eight Advanced Mycology Diagnostic research centres (AMDRCs) covering zones of the country, the details of which are given in Figure S1. The ICMR headquarters (Hqrs.), New Delhi, is the nodal coordinating centre for planning, funding, and providing all logistic support for MycoNet centres across the country. ICMR–National Institute of Medical Statistics (NIMS) joined the network to develop a digital database for ICMR MycoNet. The AMDRCs across India were invited to participate in the registry, each playing a crucial role in contributing data to the database. Here, we propose a step-by-step tool to develop such a comprehensive database, which is also depicted in Figure 1. A graphical example for adding data to the registry is shown in Figure S3.

### 2.1. Planning and Development

#### 2.1.1. Identify the Purpose and Requirements of the Database Registry

Facilitating the acquisition of standardized data is one of the primary requirements of clinical data management. The need for systematic clinical data management stems from the complexity of fungal infections, which can vary in their manifestations, resistance patterns, and responses to treatment. Data mining, which is increasingly used in medical practices, relies on the data stored in the data management system for its effective implementation. Simultaneously, potential relationships or patterns within the data can be sought that acquire valuable insights and/or underlying conditions or comorbidities relative to invasive fungal diseases, thereby ensuring the validity of the current diagnostic and treatment methods being adopted. However, the lack of such systems, particularly in large and multicentric studies, may lead to manual data management. This may lead to data quality concerns and security risks, thereby not only jeopardizing the confidentiality of the research data but also increasing the threat to human lives. Also, for national priority projects, it is important to analyse real-time data for programmatic implementation, which can only be possible with a digital data management system.

#### 2.1.2. Identify the Stakeholders and Their Roles in the Development Process

The database development process involves various stakeholders, each contributing unique expertise and perspectives. Primary stakeholders include healthcare professionals, database developers, and data managers as they provide domain knowledge, clinical expertise, and insights into the data collection protocol. To develop this database registry, the Technical Advisory Committee (TAC) comprising subject area experts oversaw the development of the protocol, granting approval for the standardized study protocol and the CRF. They ensured that the database captured relevant and accurate clinical information to support evidence-based decision making and research. The developer is responsible for designing and implementing the technical infrastructure, ensuring data security, and maintaining the database’s functionality. The designated individuals from each AMDRC can then digitally input data into a generated CRF, which is reviewed and verified by the Principal Investigators (PIs). After internal verification, data will be submitted to the central data management team for external verification as well as data cleaning.

Hence, developing a clinical registry database on IFIs necessitated collaboration between mycologists, infectious disease specialists, epidemiologists, biostatisticians, and data scientists.

#### 2.1.3. Frame a CRF

The multidisciplinary team worked together to design a comprehensive data collection protocol in the form of a CRF, ensuring patient privacy and data security in compliance with ethical guidelines and regulatory requirements set forth by the Indian Council of Medical Research (ICMR) and other relevant bodies. The CRF records all positive cases of IFIs, irrespective of their immune status (whether immunocompromised or immunocompetent) admitted to the ICUs of the participating AMDRCs. For case definitions of IFIs, we included proven and probable cases as per EORTC/MSG (proven cases) or Blot criteria and modified EORTC/MSG (probable cases), as given in Table 1. CRF was meticulously designed to accommodate various data points, ensuring that it captured all relevant information related to IFIs while remaining user-friendly for healthcare providers. It encompassed details on patient demographics, underlying comorbidities, diagnostic methods, antifungal therapies, and clinical outcomes and was divided into six sections (Figure S2). Details on the data elements for each section are given in Table S1. User-friendly forms, data entry prompts, and dropdown menus simplify data inputting for the data entry process and minimize errors (Figure S4).

#### 2.1.4. Data Collection and Completeness

In-house data collection was conducted at each AMDRC according to the CRF. The PI networked with clinicians and ICUs to facilitate the data collection. The centres also uploaded the detailed data of each identified fungus and their AMR profiles. This dataset was also used to create an online strain repository. The participating centres nominated dedicated personnel responsible for data entry, who underwent rigorous training to ensure accurate and consistent data input. Ongoing training and regular communication with participating centres were conducted to ensure complete and accurate data collection, reinforcing the importance of comprehensive data entry.

#### 2.1.5. Database Design Considerations

Designing a database structure targeting fungal infections involves a meticulous and thoughtful approach to defining the architecture, relationships, and data elements that will constitute the database’s framework. Selecting the database model is the most important consideration in this phase. Depending upon the complexity of this infection, we chose a relational database model, allowing for structured data organization and efficient data retrieval. As the next step, we thoroughly analysed the types of data collected through a CRF. This included patient demographics, medical histories, diagnostic tests, treatment regimens, and outcomes. Each entity was then dissected into attributes that captured specific data points, ensuring granular and organized representation. Establishing relationships among entities was also fundamental for upholding the integrity of the database, achieved by implementing primary and foreign keys. It helped in associating patient details with specific instances, fostering a unified data environment, preserving data uniformity, and facilitating seamless data retrieval processes. Additionally, designing the database involved anticipating potential queries and analysis requirements, which was accomplished through the integration of indices and optimization of data structures. This phase is crucial in ensuring that the database can effectively capture, store, and manage diverse clinical data related to fungal infections.

#### 2.1.6. Develop the Clinical Data Management System

The development phase translates the designed database structure into a functional and accessible clinical database tailored to fungal infections. The entire prototype was crafted using the PHP programming language, with data storage facilitated by MySQL as an appropriate database management system (DBMS). The MD5 algorithm implemented in the database masks the credentials for each AMDRC, ensuring secure and encrypted storage and safeguarding the database from unauthorized access. In a nutshell, the database development utilized an open-source database management system, web designing, and user interface standards.

#### 2.1.7. Field Testing of the CRF

CRFs have five different segments: epidemiology, clinical presentation, diagnosis, treatment, and outcome. There are a few mandatory fields that have to be filled to enable access to the subsequent question. A total of 100 CRFs with positive IFI cases were completed for field testing to finalize the CRF. The CRF was modified based on inputs from field testing. The final CRF was locked after satisfied feedback was received from PIs and experts. A digital version of the CRF was prepared and uploaded on the MycoNet website.

#### 2.1.8. Data Validation and Quality Assurance

Multiple data validation rules and quality assurance checks were implemented for all the fields in each section of the CRF to maintain the integrity and accuracy of the registry data. The multidisciplinary team was vigilant in verifying data entries, cross-referencing with source documents when necessary, and addressing inconsistencies. These ongoing data checks and quality control measures were conducted regularly both during and post-development.

#### 2.1.9. Visualizing Data Dynamics

As a part of the database development process, a dashboard was developed to provide a snapshot of critical information and performance metrics of the data from each participating centre. The registry employs a web-based data visualization platform, with all the real-time data implemented using Chart.js (version 4.3.3), an open-source JavaScript library for data visualization. JavaScript (https://developer.mozilla.org/en-US/docs/Web/JavaScript/, accessed on 2 November 2023) is a high-level client-side programming language and is one of the core technologies of the World Wide Web. The primary goal of dashboard development was to offer real-time data visualization that highlights trends, patterns, and correlations in different centres across the country. By integrating data from diverse sources, including patient records, laboratory results, and treatment regimens, dashboards provide a holistic view of the impact of fungal infections. These dynamic visualizations transform raw data into intuitive visuals, enabling users to identify noteworthy trends effortlessly.

### 2.2. Maintenance and Sustainability

The database registry is the primary repository for all collected data. The system is hosted on secure servers with firewall safety to prevent hackers from attacking data centres. Additional features include vulnerability assessment services to identify security vulnerabilities, VPNs to provide secure access to internal resources, and encrypted data transmission to safeguard patient information. A centre-wise ID and password were generated for data entry. Each centre was provided two sets of IDs and passwords, one for the data entry operator and another for the PI. Data validation is conducted by the PI. Only authorized personnel from each participating centre have controlled access to input and review data. ICMR-NIMS has overall responsibility for the monitoring, cleaning, and periodic analysis of the central database. The maintenance and sustainability of a database developed for fungal infections are critical components to ensure the system’s reliability, accuracy, and continued usefulness. The following components comprise the essential measures consistently implemented:

#### 2.2.1. Regular Database Updates

This encompasses incorporating new patient data and treatment outcomes. By capturing the latest insights, the database remains a relevant and valuable resource and ensures that the end users and healthcare professionals have access to the most recent information to direct their patient care decisions.

#### 2.2.2. Back up the Database Regularly

Regularly backing up the database, both on online servers as well as offline locations, is a fundamental practice in order to store the data securely. Backups provide a safety net against data loss due to technical failures, human errors, or unforeseen incidents. Having up-to-date backups ensures that, in the event of a system failure, data can be recovered promptly without compromising research continuity.

#### 2.2.3. Monitor the Database for Security and Performance Issues

Vigilant monitoring of the database’s security and performance helps identify any potential vulnerabilities or breaches that could compromise patient data. Performance monitoring ensures that the database operates efficiently, delivering timely access to information, and allows for swift remediation, maintaining the database’s reliability.

#### 2.2.4. Resolve any Issues That Arise

Communication with participating centres is conducted at regular intervals to resolve any issues that arise promptly, which is integral for sustaining the database’s functionality. Whether it is addressing technical glitches, data discrepancies, or security concerns, a responsive approach to issue resolution is followed for uninterrupted access to critical information. Immediate action contributes to user trust and maintains the credibility of the database as a reliable resource.

## 3. Discussion

The prevalence of fungal infections is on the rise globally, possibly attributed, at least in part, to the growing number of vulnerable patients susceptible to otherwise rare fungal infections. The complexity of fungal infections and the necessity to identify risk factors linked to the development of IFIs lie in assisting clinicians in achieving earlier diagnoses, a vital aspect of disease management, and hence has necessitated the development of a central system, moving beyond the confines of manual data management and small-scale databases. Since data management is the most crucial part of multicentric studies, there are various fungal pathogen databases and networks that play a critical role in tracking, monitoring, understanding, preventing, and managing fungal infections. For example, the CGD database funded by the National Institute of Dental & Craniofacial Research at the U.S. National Institutes of Health provides a wealth of genomic sequence data as well as gene and protein information for *Candida albicans* and related species [15]. *Candida albicans,* a common commensal organism of healthy individuals, can cause debilitating mucosal infections and life-threatening systemic infections, especially in immunocompromised patients. It also serves as a model organism for the study of other fungal pathogens aiding researchers in understanding the genetics and biology of these pathogens. The Fungal MLST database makes publicly available the data generated from MLST, a primary typing approach using the partial sequence analysis of seven to ten housekeeping genes to study the epidemiology of diverse fungi [16]. Similarly, FUNGINOS conducted a 15-year study of candidemia from 2004 to 2018 in Switzerland. The study included the hospital-based incidence of candidemia, *Candida* species distribution, antifungal susceptibility, and consumption [17]. The ECMM operates across Europe and gathers IFI data from participating centres [18]. TRANSNET, a consortium of 23 U.S. transplant centres, focuses on monitoring IFIs in transplant recipients and understanding the disease burden in the U.S. [19]. The ASID Mycology Interest Group Registry ANZMIG specifically focuses on IFIs in Australia and New Zealand [20]. FungiScope™—Global Emerging Fungal Infection Registry is a global registry that aims to track emerging fungal infections and supports efforts to control their spread worldwide. The registry has partners from 66 countries and collects data on epidemiology, pathogen biology, and clinical course of IFIs [21]. Fung-I-Reg, a pan-India web-based registry, focuses on fungal infections namely *Aspergillus* species, Mucorales, as well as other rare fungi [22]. However, all of these existing registries are either pathogen-specific or fragmented across different regions, institutions, and countries. This fragmentation can make it challenging to integrate and standardize data, hindering comprehensive research efforts in understanding the disease. 

In the present study, the data management system was developed and implemented to address the complexities associated with not only specific but also complete fungi. Most data management systems involve four main components. The first aspect of the system includes a management module for designing the database, creating CRFs, involving researchers and research centres, maintaining security, and controlling user access. The IFI data entry is conducted through the graphical user interface that makes up the second component, and the third component includes the PI of the respective centre verifying and validating the data entry. It incorporates various data quality control measures and validation rules tailored to specific fields. The fourth component comprises both a dashboard and a reporting module, facilitating the generation of essential reports concerning the data and the progress of the study [23]. All of these factors were considered in the database developed in the present study. The required data elements to design a CRF were categorized into socio-demographic characteristics, clinical presentations, host and risk factors, diagnostics, fungal pathogen distribution, treatment, and outcomes. The main functionality of the system lies in the aspects of designing CRFs, data management, data quality and control, and confidentiality. OpenClinica and REDCap have also considered similar rules [24,25,26]. While other systems, such as Ez-Entry, ObTiMA, and OnWARD, defined equal validation rules for all fields, our system has different validation rules for each field that we defined in the CRF [27,28,29].

One of the key outcomes of our clinical database management system is its ability to facilitate standardized data collection. By employing well-defined data elements and structured data entry forms, we ensured consistency in data input, minimizing errors and enhancing the quality and reliability of the data. This standardization enables seamless comparisons and analyses across different studies, institutions, and geographical regions, contributing to a more comprehensive understanding of fungal infections’ epidemiology and treatment outcomes. Reporting and extracting data are two of the major components of a clinical data management system. All the extracted data are readable by various software and libraries like pandas, SPSS, and STATA and are reported for further detailed analysis and investigations. The database’s real-time data visualization and exploration features emerged as a powerful tool for data monitoring and analysis. They enable the identification of trends, patterns, and anomalies promptly. Treatment efficacy is one of the key outcomes in the study of fungal infections. An illustration of the success rates of different antifungal treatments allows healthcare professionals to evaluate which interventions yield the most favourable outcomes. This real-time insight will generate India-specific data, enabling clinicians to tailor treatments based on the most effective approaches in our population. This aspect of the system empowers clinicians to make informed decisions and researchers to detect emerging trends or treatment responses swiftly. In the era of different global threats like climate change, pandemics, and antimicrobial resistance (AMR), a user-friendly, secure digital database is one of the important research tools for the visualization of real-time data and the quick analysis of disease trends, which would help quick decision making during an emergency. It will also reduce the manual errors of data entry and analysis.

However, while our clinical database management system demonstrates several strengths, there are also areas for improvement. The developed database system was critically evaluated by the clinicians and supervisors involved in the study. Routine feedback on data entry and the graphical user interface (GUI) was obtained through individual sessions with each AMDRC. In terms of usability, most of the problems were related to the user interface design or ambiguity of the clinical terms used in the interface. Ensuring complete and accurate data collection proved challenging due to varying healthcare practices across different institutions. Despite the rigorous quality control measures, data entry errors may still occur, necessitating constant vigilance and dedicated efforts to maintain data accuracy. However, centres went through extensive hands-on training program from PGI, Chandigarh, who are the EQAS and training partner in this program. The issues could also be addressed through the use of standard case definitions, clinical terminologies, and diagnostic and treatment algorithms across centres, which will be implemented via capacity-building programs of the site investigators. The errors related to data will be resolved by including frequent training of the data entry operators, validation checks, and regular data cleaning. Additionally, the system’s scalability needs to be carefully considered to accommodate potential increases in data volume and user demands as the database gains wider adoption.

## 4. Conclusions

The insights gained from the registry data will improve invasive fungal disease surveillance in India, which in turn will empower policymakers to identify areas with high disease burden and prioritise resource allocation. This registry will enable the optimization of diagnostic and treatment regimens and early identification of drug resistance patterns, which will improve treatment outcomes. Further expanding the scope of the registry to include additional tertiary healthcare centres covering diverse geographical regions will further enhance the scope of the clinical registries and, hence, will create a more comprehensive and interconnected knowledge base on fungal infections. For example, currently, fungal disease surveillance under the National Centre for Disease Control (NCDC) is in the incipient stage in India [30]. The integration and utilization of the clinical registry database along with the Integrated Disease Surveillance Project [31] and antimicrobial resistance (AMR) containment program [32], spearheaded by the NCDC and the Ministry of Health & Family Welfare (MOHFW), will aid policymakers to streamline efforts on improving fungal disease surveillance and monitor antifungal resistance patterns in India.

## Figures and Tables

**Table 1:** Case definition of IFI cases as per EORTC/MSG (proven cases) or Blot criteria and modified EORTC/MSG (probable cases).

S. No.	Category	Causative Agent	Criteria	Details
1.	Proven	For all IFIs	EORTC/MSG criteria, 2019	Table S2
2.	Probable	Invasive Pulmonary Aspergillosis (IPA)	Blot criteria 2012, 19	Table S3
		Other clinical forms of Aspergillosis or other moulds, yeasts, or yeast-like fungi	Modified EORTC/MSG based on Blot’s clinical algorithm (compatible clinical/imaging/mycology findings in different host populations)	Table S4

## Data Availability

Data are contained within the article and Supplementary Materials.

## Supplementary Materials

The following supporting information can be downloaded at: https://www.mdpi.com/article/10.3390/jof10010042/s1, Figure S1: ICMR Advanced Mycology Diagnostic and Research Centres; Figure S2: Overview of the CRF in the database registry; Figure S3: Graphical example of the interface for adding data to the registry; Figure S4: Snapshot showcasing the data entry prompts; Table S1: Overview of data elements in the CRF; Table S2: EORTC/MSG criteria^52^, 2019; Table S3: BLOT criteria^54^ 2012, 2019; Table S4: Modified EORTC/MSG based on Blot’s clinical algorithm.
