# Supplementary material for: The Development of a Clinical Registry Digital Database on Invasive Fungal Infections in India: Advancing Epidemiological Understanding and Patient Care
Source: J Fungi (Basel). 2024 Jan 5;10(1):42. doi: 10.3390/jof10010042 (PMC10817322; doi:10.3390/jof10010042)
Supplement: Supplementary file 1 [file jof-10-00042-s001.zip › jof-2754956-supplementary.pdf]

Supplementary Materials:

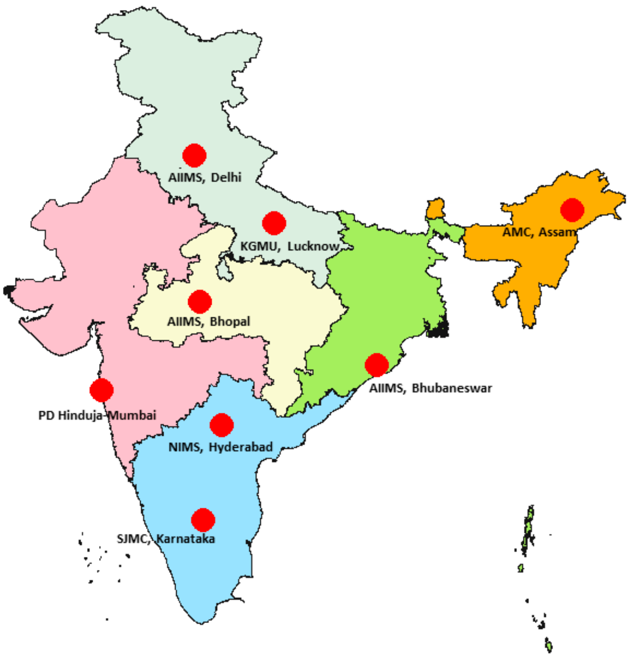

Figure S1. ICMR Advanced Mycology Diagnostic and Research Centres.

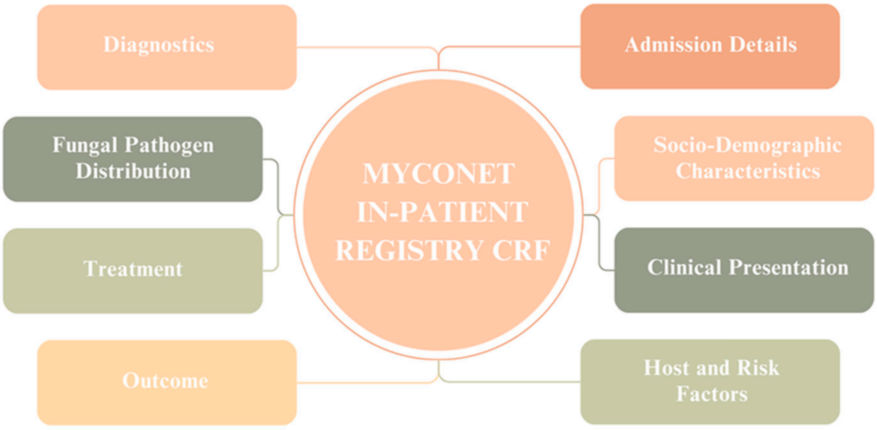

Figure S2. Overview of the CRF in the database registry.

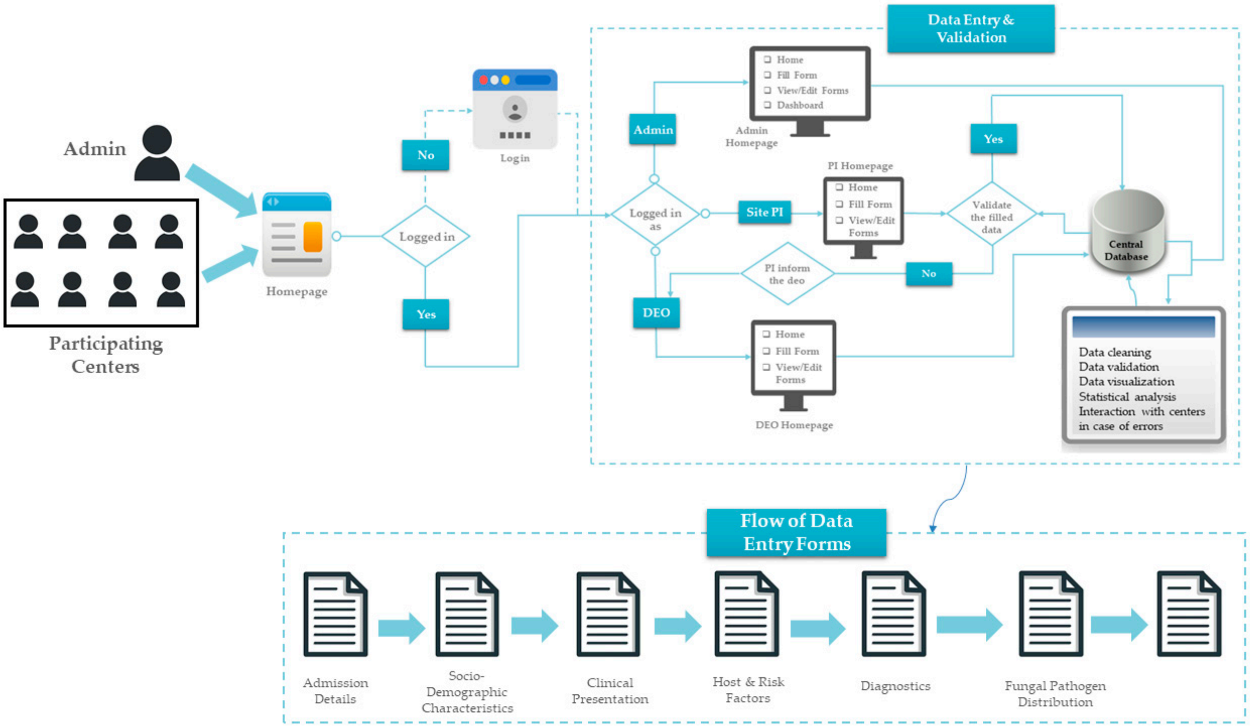

Figure S3. Graphical example of the interface for adding data to the registry.

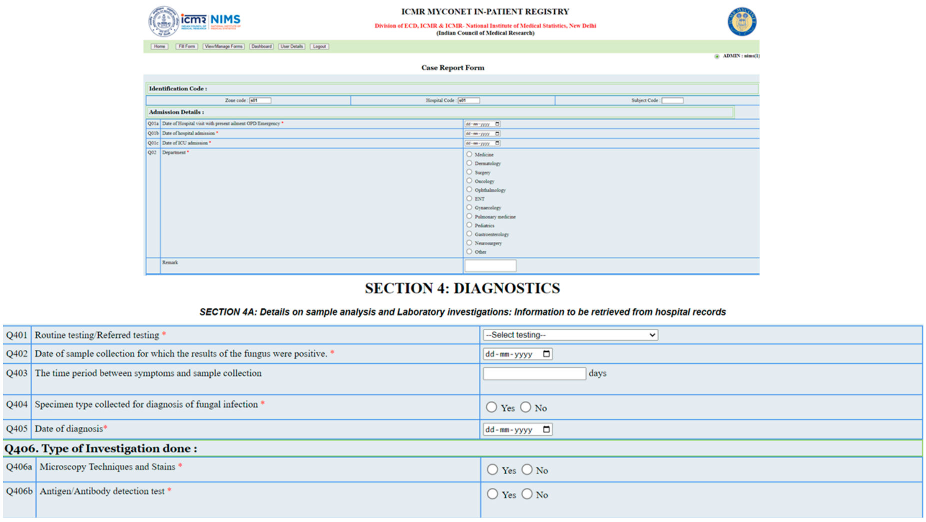

Figure S4. Snapshot showcasing the data entry prompts

Table S1. Overview of data elements in the CRF.

S. No.	Type of Data	Data Elements
**1.**	**Admission Details**	1. Date of hospital visit<br>2. Date of hospital admission<br>3. Date of ICU admission<br>4. Department
**2.**	**Section 1**	1. UHID/Pt. ID

	**Socio-Demographic Characteristics**	2. Date of birth<br>3. Age<br>4. Gender of the participant<br>5. Address<br>6. State<br>7. District<br>8. Pin code<br>9. Contact number<br>10. Housing Structure<br>11. Patient's weight<br>12. Patient's height<br>13. Head circumference
**3.**	**Section 2**<br>**Clinical Presentation**	Clinical symptoms according to the main sites of fungal infection<br>1. Pulmonary Involvement<br>2. Rhino-cerebral/Orbital Involvement<br>3. Central nervous system involvement<br>4. Skin and soft-tissue involvement (for all IFIs/Mycetoma/Chromoblastomycosis)<br>5. Eye involvement<br>6. Gastrointestinal system involvement<br>7. Disseminated infection<br>8. Joint involvement<br>9. Date of transfer from ICU to any other ward<br>10. H/o prior admission
**4.**	**Section 3**<br>**Host and Risk Factors**	1. Requirement of non-invasive ventilation (NIV)<br>2. Date of start of NIV<br>3. Date of completion of NIV<br>4. Duration of NIV before onset of IFI<br>5. Requirement of invasive ventilation (IV)<br>6. Date of start of IV<br>7. Date of completion of IV<br>8. Duration of IV before onset of IFI<br>9. Date of symptom onset<br>10. Underlying medical conditions<br>11. Known risk factors<br>12. Whether received corticosteroid

		13. Type of steroid<br>14. Any immunosuppressive drug received (ID)<br>15. Any concomitant infection
**5.**	**Section 4A**<br>**Diagnostics**	1. Type of testing<br>2. Date of sample collection (positive fungus)<br>3. The time period between symptoms and sample collection<br>4. Specimen type collected for diagnosis of fungal infection<br>5. Date of diagnosis<br>6. Type of investigation done (Microscopy techniques and stains)<br>7. Antigen/Antibody detection test available<br>8. Whether fungal culture positive<br>9. If antifungal susceptibility testing is done<br>10. Methods of AFST<br>• Micro broth dilution Colorimetric<br>• Sensititre Yeast One<br>• Macrobroth dilution<br>• VITEK-2<br>• E-Test<br>• Agar dilution<br>• Other<br>11. If direct PCR from the clinical sample is done<br>12. Therapeutic drug monitoring<br>13. Histopathology<br>14. Radiology/Additional tests<br>15. Laboratory investigations<br>16. Highest random blood glucose levels during hospital stay<br>17. Highest ferritin level during hospital stay<br>18. Highest serum IL-6 during hospital stay<br>19. The period between symptoms and diagnosis<br>20. The period between sample collection and diagnosis
**6.**	**Section 4B**<br>**Fungal Pathogen Distribution**	Fungal pathogen type (species level)<br>1. Ascomycetes<br>2. Zygomycetes<br>3. Basidiomycetes
**7.**	**Section 5**<br>**Treatment**	1. Antifungal strategies<br>2. Type of antifungal therapy

		3. Antifungal drug<br>4. Drug-related adverse events<br>5. Surgical procedures<br>6. Adjunctive therapy
**8.**	**Section 6**<br>**Outcome**	1. Date of outcome<br>2. Outcome<br>3. Cause of death<br>4. Duration of stay for present illness in the hospital<br>5. IFI-attributable mortality<br>6. Was the IFI identified as hospital-acquired infection?<br>7. Was the isolate yeast/mold/mixed/other<br>8. Was nosocomial outbreak analysed?<br>9. Autopsy (if available)<br>10. Final diagnosis at the time of discharge/otcome<br>11. Final diagnosis based on CRF

Table S2. EORTC/MSG criteria[52], 2019

Causative agent	Requirement	Details
IPA<br>or<br>Other molds	• Sterile material<br>• Needle aspiration or sterile Lung biopsy or blood	**1. Histopathologic, cytopathologic, or direct microscopic** examination of a specimen in which hyphae are seen accompanied by evidence of associated tissue damage.<br>**2. Culture on sterile material,** excluding BAL fluid, a paranasal or mastoid sinus cavity specimen, and urine<br>**3. Blood culture** for *Fusarium* species only<br>**4. Serology**: Not applicable<br>**5. Molecular diagnosis:** PCR +ve with DNA Sequencing along when hyphae seen in formalin fixed paraffin embedded sections (FFPE)
Candidemia<br>or<br>Cryptococcosis<br>or<br>Yeast like fungi	Sterile specimens/ Blood/CSF	**1. Histopathologic, cytopathologic, or direct microscopic examination:** Sterile specimens obtained by needle aspiration or biopsy from a normally sterile site (other than mucous membranes) showing budding yeast cells or with pseudo hyphae/hyphae<br>**2. Recovery of a yeast by culture of a sample obtained by a sterile procedure** (including a freshly placed [<24 hours ago] drain) from a normally sterile site

		**3. Blood culture** that yields yeast (eg, *Cryptococcus* or *Candida* species) or yeast-like fungi (eg, *Trichosporon* species)<br>**4. Serology:** Cryptococcal antigen in cerebrospinal fluid or blood<br>**5. Molecular diagnosis:** PCR +ve with DNA Sequencing along when yeasts are seen in FFPE
Pneumocystis		**Detection of the organism microscopically in tissue, BAL fluid, expectorated sputum** using conventional GMS staining or immunofluorescence staining<br>**Culture/Serology/Molecular:** Not applicable
Endemic mycoses		**Histopathology or direct microscopy** of specimens obtained from an affected site showing the distinctive form of the fungus<br>**Culture:** Recovery by culture of the fungus from specimens from an affected site Blood culture that yields the fungus<br>**Serology/Molecular diagnosis:** Not applicable

Table S3. BLOT criteria[54] 2012, 2019

Causative agent	Requirement	Details
**Probable IPA**	• All 4 condition should be meet i.e.,<br>1. Mycological criteria<br>2. Radiology<br>3. Clinical presentation<br>4. Host factors (underlying conditions)<br>• Higher likelihood of Probable IPA if all 4 condition meet,<br>• However, in absence of disease severity /underlying condition suggestive mycology finding and radiologic feature should not be neglected in diagnosis of IPA	**1. Mycological criteria (any of the direct or indirect test positive)**<br>**Entry criteria**<br>a) **Direct test (either microscopy or culture positive or both)**<br>• Respiratory tract samples (preferably lower tract specimens) i.e. BAL/ ET aspirate / Sputum etc.<br>• Cytology/Direct microscopy – Positive<br>• Culture (Qualitative/Semi-quantitative) -Positive<br>b) **Indirect tests**<br>• Serum: GM-positive (>0.5 or higher)<br>• BAL fluid: GM-positive (>0.5 or higher)<br>• LFD-test: positive<br>• Aspergillus PCR positive
		**2. Radiological features (any one of the following)**<br>• Dense, well circumscribed lesion(s) with or without a halo sign<br>• Air-crescent sign<br>• Cavity formation<br>• ARDS like images or<br>• nonspecific infiltrates<br>• consolidation<br>• Abnormal imaging on chest X-ray
		**3. Clinical presentation (any one of the following)**

		• Severe respiratory failure and need for extra-corporal membrane oxygenation (ECMO)<br>• Influenza/Covid-19 or other viral infection<br>• Fever refractory to at least three days of appropriate antibiotic therapy<br>• Recrudescent fever after a period of defervescence of at least 48 h while still on antibiotics and without other apparent cause<br>• Pleuritic chest pain<br>• Pleuritic rub<br>• Dyspnea<br>• Hemoptysis<br>• Worsening respiratory insufficiency in spite of appropriate antibiotic therapy and ventilatory support<br>• Sepsis/multi-organ failure<br>• ARDS<br>• Pneumonia<br>• Higher SOFA (>5)/Apache score
		**4. Host factors/underlying conditions (One of the following):**<br>**Immunocompromised state**<br>• Neutropenia,<br>• Haematological or oncological malignancy<br>• Glucocorticoid treatment,<br>• Congenital or<br>• Acquired immunodeficiency syndrome<br>**Other underlying conditions**<br>• Hepatic cirrhosis<br>• HIV/AIDS<br>• Chronic obstructive pulmonary disease (COPD)<br>• Chronic alcohol abuse<br>• Diabetes mellitus,<br>• Chronic heart failure,<br>• Chronic renal failure<br>• Haemodialysis etc.

Table S4. Modified EORTC/MSG based on Blot's clinical algorithm

Causative agent	Requirement	Details
Other clinical forms of Aspergillosis or other Molds or Yeasts or Yeast like fungi	• Presence of all of the following, as described by Blot et.al. 2012, 2019 but not limited.<br>• Variable with Causative agent, clinical disease presentations	1. **Risk factors** as described by Blot et.al. 2012, 2019 but not limited<br>2. **Compatible clinical features** including acute localized pain (including pain radiating to the eye), Nasal ulcer with black eschar, Focal lesions on imaging etc.<br>3. **Imaging:** as described by Blot et.al. but not limited including mycotic aneurism, bird's nest sign, multiple large nodule, pneumothorax, bull's-eye lesions in liver/spleen/brain etc.<br>4. **Mycology evidence:**<br>a. **Either Direct microscopy or culture positive** (preferably both) from **any non-sterile sites**<br>b. **Serology: β-D-glucan** (Fungitell) ≥80 ng/L (pg/mL) detection in **>2 consecutive serum samples** provided other etiologies have been excluded (for Candida/pneumocystis)
